# WeChat: a new clinical teaching tool for problem-based learning

**DOI:** 10.5116/ijme.5708.e5c4

**Published:** 2016-04-25

**Authors:** Furong Zeng, Guangtong Deng, Zhao Wang, Longfei Liu

**Affiliations:** 1 Xiangya Medical School ,Central South University, China; 2 Xiangya hospital, Central South University, China

**Keywords:** WebChat, clinical teaching, problem-based learning

## Introduction

Problem-based learning (PBL) is an instructional student-centered approach which utilizes carefully constructed real-world, ill-defined problems as a context for students to extend their prior experiences and knowledge through discussion and self-directed and collaborative learning that fosters the development of problem-solving and reasoning skills.^1^ Compared with traditional lecture-dominant teaching and learning approaches, PBL prompts students to actively engage not only in knowledge construction and develop competencies across multiple contexts,^2^^,^^3^ but also in self-directed learning and general work-related skills.^4^ PBL has a large contribution to medical education, but it has limitations. It is considered less effective than traditional learning methods because it is time-consuming.^5^ Other obstacles to the popularity of PBL include a predominating uncertainty about the breadth and depth of learning without a syllabus, concerns about overlooking things, and the high self-motivation required.^6^ So implementations of PBL is highly desirable.

It is well known that Facebook and Twitter are introduced into medical education in colleges and universities in the western countries,^7^ achieving great success. Recently, Chinese medical educators are actively exploring new teaching methods to adapt to the social development. New media, WeChat,^8^ have been widely utilized in medical schools and exert a positive influence. WeChat is the most popular platform visited daily by university students in China. It is currently recognized as the best social networking site used by people of all ages and professions in China. WeChat is connecting more than a half billion Chinese people now. Apart from free chat, WeChat’s large-scale network platform hosts a vast amount of user-generated data, including text, voice call, video, and images. Group chat is perfect for professional online discussion. Its characteristics of convenience, promptness, and good cross-platform support enrich and simplify the communication model to offer WeChat application users communication that is more prompt.^9^ WeChat, owing to its immediate communication and source sharing, break the spatial and temporal limitations and change the teaching mode of the traditional classroom, making communication more convenient.

### The teaching mode of WeChat combined with PBL

Ethics Review is used for a ongoing prospective study comparing WeChat-PBL and PBL. It is not necessary to the present descriptive study. The teacher divides all students into several WeChat groups with 3-4 students per group. After obtaining written agreement from patients, the teacher collects their clinical data, including basic information, chief complaint, symptoms, signs, and laboratory examinations. These data are presented in words. Imaging data and surgery materials are presented in images and video, respectively. The teacher delivers the materials and questions to the WeChat groups before class. The students research this material individually and then discuss the questions with each other in their small WeChat group. They can communicate and exchange ideas with their WeChat group colleagues to solve the problems. One representative of each group shows their discussion and conclusions in a slide show to the classroom. The teacher makes comments about their work. Students can further discuss their experiences after class to enhance the teaching effects.

### Advantages of this teaching model

#### Abundant resources

In PBL, teachers and students are able to share all kinds of learning materials, including the latest developments published in journals, papers, books, and a great variety of images, voice recordings, and surgery videos. Using WeChat, students can obtain greater overall knowledge, which compensates for the “missing” educational elements of PBL. Furthermore, the WeChat platform offers more communication opportunities for teachers and students. Teachers can also share their new ideas and experiences outside the classroom environment.

#### Improved learning enthusiasm

In WeChat groups, students discuss problems and express their opinions. Compared with the traditional “cramming” system of study, WeChat discussions encourage students to interact to reinforce their learning while also accomplishing their tasks independently and actively. This system also works for students with poor self-motivation. The images and videos are so vivid that students are able to understand the material thoroughly. If students have any questions, they can leave a WeChat message and their teacher or colleagues will respond. Teachers are able to teach students according to their aptitude and thus improve learning efficiency.

#### Convenience

Teachers can deliver learning materials without limitations of time and space. In their spare time, teachers can pick up typical cases, create questions, and then send it to the WeChat groups before class. The system allows small fragments of time to be used in learning and resolves the “time-consuming” problem of PBL. Students research papers individually and discuss them in their WeChat groups. These discussions help them to solve problems rapidly after class. The WeChat–PBL system pushes traditional teaching methods to a higher level.

### Disadvantages of this teaching model

#### Poor continuity

WeChat requires Internet access and Wi-Fi is not available everywhere, especially in some universities. The breaks in continuity of access create a limitation of flexibility in time and space, and students lack an objective environment for successful learning. Consequently, network use is degraded, leading to lower efficiency. There is still a long way to go before wireless networks are ubiquitous in classrooms, dormitories, or hospitals.

#### Privacy disclosure

Patient privacy must be protected when using surgery video materials and their use requires the agreement of patients. However, WeChat allows patient information to be rapidly distributed, and patient data may be easily leaked. To reduce this risk, we removed the identity information from images, videos, and other visual materials after obtaining agreement from the patients. Students were requested to avoid posting the materials on the Internet outside their WeChat group.

#### Poor social skills

Context based on the network is vague and implicit to a great extent and the cooperative principle and strategy applicable to the face-to-face interactions is broken with long-term WeChat communication. Students may become introverted and indifferent if they are addicted to WeChat and talk little with each other in real life because of their poor social skills. These poor social skills would become an obstacle to their doctor–patient relationships in later life.

## Conclusions

Using the WeChat–PBL platform in medical teaching emphasizes interactive, individual, and computer multimedia teaching styles. As a new platform, WeChat stimulates students’ learning interest and enthusiasm, improves their self-directed learning ability, enhances the collaboration with peers, and then promote the education reform. With this platform, teachers can teach students according to their individual aptitudes and make great progress. The educational use of WeChat is undeniably underexplored.

With the advent of the information age, new media as a communication tool will accelerate further advancement of medical teaching. Future medical education models are likely to undergo large changes. Medical education has become globalized, and there are new challenges emerging. How to make better use of WeChat and combine it with the traditional teaching well still needs exploration. Therefore, both medical educators and students need to change inherent thinking to advance with the times and take the initiative to adjust to the development of science and technology, thus innovating medical education methods.

### Conflicts of Interest

The authors declare that they have no conflict of interest.
